# Survival and medical utilization of children and adolescents with prolonged ventilator-dependent and associated factors

**DOI:** 10.1371/journal.pone.0179274

**Published:** 2017-06-19

**Authors:** Szu-Chi Pai, Pei-Tseng Kung, Wen-Yu Chou, Tsunghuai Kuo, Wen-Chen Tsai

**Affiliations:** 1 Department of Health Services Administration, China Medical University, Taichung, Taiwan; 2 Department of Respiratory Therapy, Show Chwan Memorial Hospital, Changhua, Taiwan; 3 Department of Health Administration, Asia University, Taichung, Taiwan; 4 Department of Chest Medicine, Show Chwan Memorial Hospital, Changhua, Taiwan; National Yang-Ming University, TAIWAN

## Abstract

Over the course of a year, more than 20,000 patients in Taiwan require prolonged mechanical ventilation (PMV). Data from the National Health Insurance Research Database for patients between 2005 and 2011 were used to conduct a retrospective analysis on ventilator dependence. The study subjects were PMV patients aged <17 years in Taiwan. A multiple regression model employing general estimating equations was applied to investigate the factors affecting the use of medical resources by children and adolescent PMV patients. A Cox proportional hazard model was incorporated to explore the factors affecting the survival of these patients. Data were collected for a total of 1,019 children and adolescent PMV patients in Taiwan. The results revealed that the average number of outpatient visits per subject was 32.1 times per year, whereas emergency treatments averaged 1.56 times per year per subject and hospitalizations averaged 160.8 days per year per subject. Regarding average annual medical costs, hospitalizations accounted for the largest portion at NT$821,703 per year per subject, followed by outpatient care at NT$123,136 per year per subject and emergency care at NT$3,806 per year per subject. The demographic results indicated that the patients were predominately male (61.24%), with those under 1 year of age accounting for the highest percentage (36.38%). According to the Kaplan—Meier curve, the 1-year and 5-year mortality rates of the patients were approximately 32% and 47%, respectively. The following factors affecting the survival rate were considered: age, the Charlson Comorbidity Index (CCI), diagnosis type necessitating ventilator use, and whether an invasive ventilator was used. This study investigated the use of medical resources and the survival rates of children and adolescent PMV patients. The findings of this study can serve as a reference for the National Health Insurance Administration in promoting its future integrated pilot projects on ventilator dependency.

## Introduction

The number of prolonged mechanical ventilation (PMV) patients in Taiwan has been increasing annually, and is currently more than 20,000 [1]. According to the Centers for Medicare and Medicaid Services in the United States (US), the term PMV patients refers to patients who have continuously relied on mechanical ventilation for more than 21 days (more than 6 hours/day) [2]. The rate of PMV incidence was 6 per 100 mechanical ventilation patients in the United Kingdom in 2002–2006 [3] and 3%–7% of mechanical ventilation patients in the US [4]. The first-year mortality rate of US PMV patients was 73% in 2013 [5]. The mortality rate of PMV patients in Taiwan was 45% in 2010 [6].

To prevent the improper use of intensive care beds, the Ministry of Health and Welfare in Taiwan established the Integrated Delivery System in 2000. When a patient has used a ventilator for more than 21 days, the patient must be transferred from the ICU to the respiratory care center. When the patient has used a ventilator for more than 63 days, the patient must be transferred to the respiratory care ward. Subsequently, the patient may receive home care after his or her condition has stabilized [7]. According to the statistical data by the National Health Insurance (NHI) Administration, in 2013 the medical expense of PMV patients was the third highest of those of the five catastrophic injuries and illnesses listed by the Ministry of Health and Welfare (NT$16.6 billion in total); a total of 22,831 patients were hospitalized, and each patient spent an average of NT$679,000 annually on medical care. Since 2013, the annual healthcare expenses of PMV patients have remained high [8].

Currently, all PMV patients in Taiwan are managed by the Ministry of Health and Welfare. Most of the child PMV patients are hospitalized in respiratory care wards and use invasive ventilators [9]. In comparison, most of the PMV patients in the United Kingdom and Italy receive home care and use noninvasive ventilators [10, 11]. Thus, child PMV patients in Taiwan receive treatment approaches differing considerably from those received by child PMV patients of other countries. Currently, Taiwan has approximately 1,000 child and adolescent PMV patients, whose use of medical resources and survival merit further examination. Therefore, this study investigated the medical services and expenses of child and adolescent PMV patients as well as the factors that affect their survival.

## Material and methods

### Study subjects

In Taiwan, the National Health Insurance Administration (NHI) separates patients with ventilator dependency into two age groups including those aged <17 years and those aged ≧17 years. The NHI stipulates that when patients under 17 years of age with ventilator dependency are required special examination and approval. In Taiwan, prolonged mechanical ventilation (PMV) patients are defined by the National Health Insurance Administration as the follows: 1. Invasive mechanical ventilation for 21 or more days; 2. invasive mechanical ventilation followed by non-invasive ventilation with a total duration of 21 or more days; 3. specific diseases which require non-invasive ventilation for 21 or more days. Invasive ventilation is referred to as conventional mechanical ventilation, which is delivered via an endotracheal tube or tracheostomy tube. In contrast, non-invasive ventilation is delivered through an alternative interface, usually a face mask.

In this study, a retrospective cohort study was conducted on child and adolescent PMV patients aged <17 years [7]. A PMV patient is defined as a person that has used a ventilator for >21 continuous days and >6 hours per day in Taiwan [2]. Newly hospitalized PMV patients aged <17 years from January 1, 2005 to December 31, 2010 were collected and followed up until December 31, 2011. Patients who were given the ICD-9-CM diagnosis codes of 518.85 or 518.81 were sampled. All patients were diagnosed as a patient with chronic PMV dependency that was one kind of catastrophic illnesses defined by the Taiwan National Health Insurance Administration in this study. This study did not include new onset patients in acute and critical but reversible status. This study was approved by the institutional review board of China Medical University Hospital (IRB number: CMUH102-REC3-105).

### Data sources

This study employed secondary data acquired from the 2005–2011 National Health Research Database and the Catastrophic Illnesses and Injuries File, which were managed and released by the National Health Research Institutes. The data sets were obtained for special requirements from the National Health Insurance Research Database managed by the National Health Research Institutes. The coverage ratio of the NHI in Taiwan over the study period was 99.68% [12]. The cases examined in this study constituted every child and adolescent PMV patient in Taiwan. In this study, all chronic PMV patients are listed as patients with catastrophic illnesses by the NHI Administration and are mostly exempt from their medical expenses [13]. Thus, medical expense burdens on such patients diagnosed with severe diseases and requiring long-term care can be mitigated.

The data contained relevant variable information from the data sets including ambulatory care and expenditure file, details of ambulatory care orders and medication file, inpatient care and expenditure file, details of inpatient care orders and medication file, and all contracted medical facilities file, registry for catastrophic illness patients file, and registry for beneficiaries file.

### Description of variables

The independent variables were the characteristics of the patients (age and sexes), their economic factor (monthly income by their parents), their environmental factor (urbanization level of residence area), their health statuses (comorbidity), their reasons for requiring ventilator use (diagnosed diseases prompting ventilation), and status of invasive ventilation use (current use/ever use or never use). The PMV patients were grouped according to their sex (male and female), their age (<1 year, 1–4 years, 5–9 years, 10–14 years, and 15–16 years), the monthly income of their parents (≤NT$17,280, NT$17,281–NT$28,800, NT$28,801–NT$36,300, NT$36,301–NT$57,800, and ≥NT$57,801), the urbanization level of residence area (Level 1, Level 2, level 3, Level 4, and Levels 5–7; Level 1 is the highest, and Level 7 is the lowest) [14], and their health statuses according to the Charlson Comorbidity Index ([CCI]; 0, 1, and ≥2) [15].

According to Wallis et al. [10], the primary diagnosed diseases of the child and adolescent PMV patients were divided into four types, namely musculoskeletal, respiratory, central nervous system, and other diseases. These diseases that required ventilation were mainly primary and secondary diseases diagnosed upon the first hospitalization in accordance with the ICD-9-CM diagnosis code. Whether the patients had ever used invasive ventilators was assessed according to their medical histories, and was defined as whether invasive ventilators had been inserted into the endotracheal tubes of the patients. The uses of medical resources were categorized as the number of outpatient visits, the number of emergency services received, the number of days of hospitalization, and the annual mean of each of the aforementioned three factors. The medical expenses were categorized as the average annual expenses of outpatient visits, emergency services, and hospitalization as well as the average annual total medical expenses and NHI expenses. Regarding the survival of the PMV patients, when a patient was not cured and no longer enrolled in the National Health Insurance Program, the patient was considered as deceased; otherwise, the patient was considered surviving.

### Statistical analysis

Frequency distributions and percentage indices were used to determine the frequencies and distributions of the aforementioned variables (i.e., sex, age, monthly income of parents, urbanization level of residence area, health status, the diagnosed disease prompting ventilation, and the status of invasive ventilation use). Means and standard deviations were employed to depict the medical services and expenses of the patients with varying independent variables (i.e., numbers of outpatient visits, emergency services, and days of hospitalization as well as related medical expenses).

Regarding the factors that affected the survival of the PMV patients, a log—rank test was conducted for a bivariate analysis on the effects of the characteristics of the patients, their economic and environmental factors, the diagnosed diseases leading to their ventilation, and their status of invasive ventilation use on the survival of the patients. A Cox proportional hazard model was incorporated to investigate the effects of the independent variables on the survival of the patients. Since whether the patients ever used the invasive ventilation had an interaction with the length of ventilator dependency in the survival analysis, in order to resolve the interaction issue, we divided the length of ventilator dependency into two periods including ≦4.5 years and >4.5 years in the Cox proportional hazard model.

To accurately measure the annual medical service use and expense of the patients, those that were examined for less than 1 year (e.g., those that were examined for an insufficient amount of time and those that died after less than 1 year of hospitalization) were excluded, resulting in a total of 547 patients being analyzed. Because the sampled data were repeatedly measured, generalized estimating equations were employed to determine inferential statistics. In other words, a multiple regression analysis was performed on the dependent variables of the patients (i.e., medical service uses and expenses in outpatient visits, emergency services, and hospitalization) and their associated factors. The Statistical Analysis System (Version 9.3, SAS Institute Inc., Cary, NC, USA) was used for statistical analysis.

## Results

**Fig 1:**
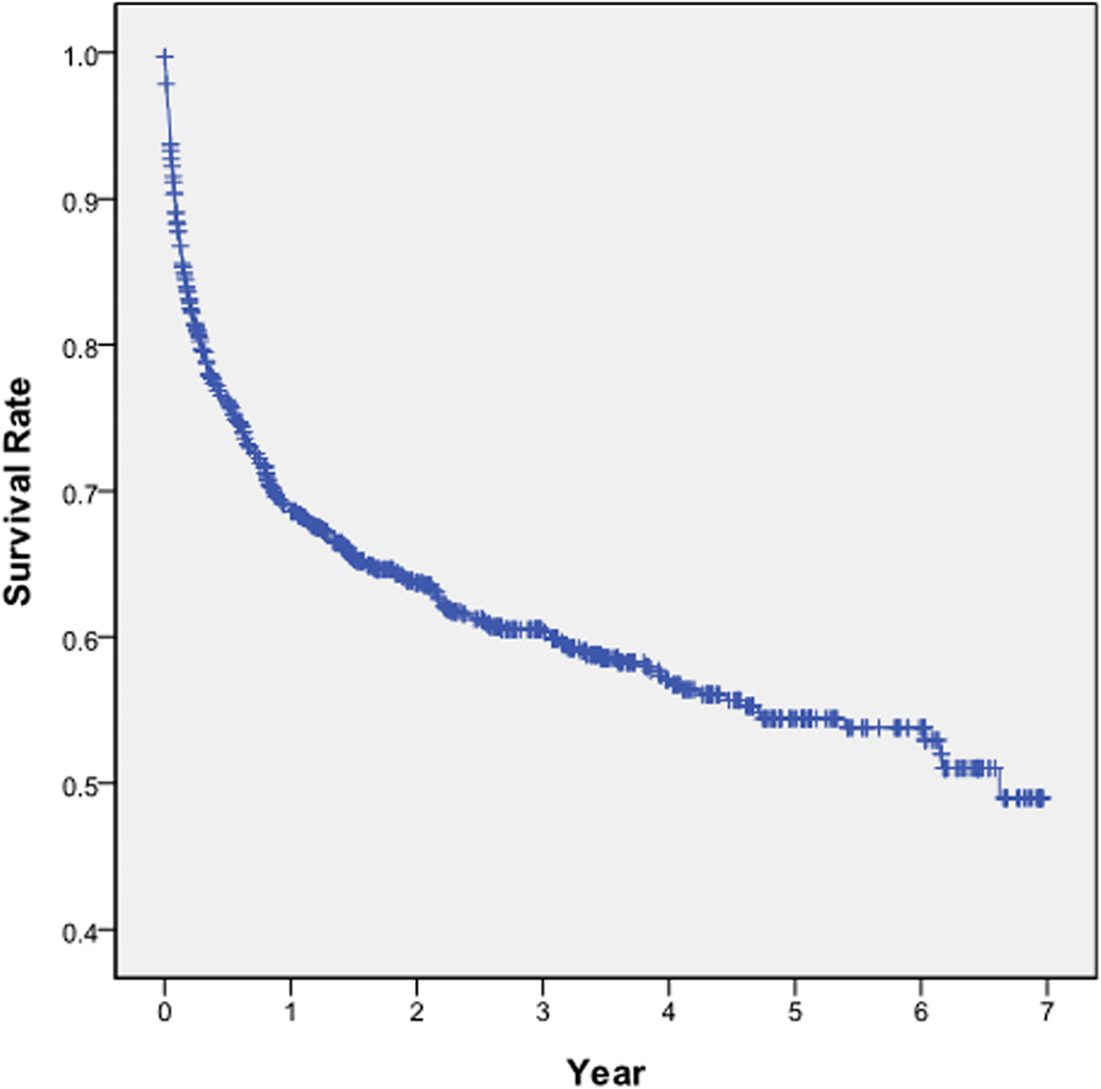
KM curve for the survival rates of the child and adolescent PMV patients.

**Fig 2:**
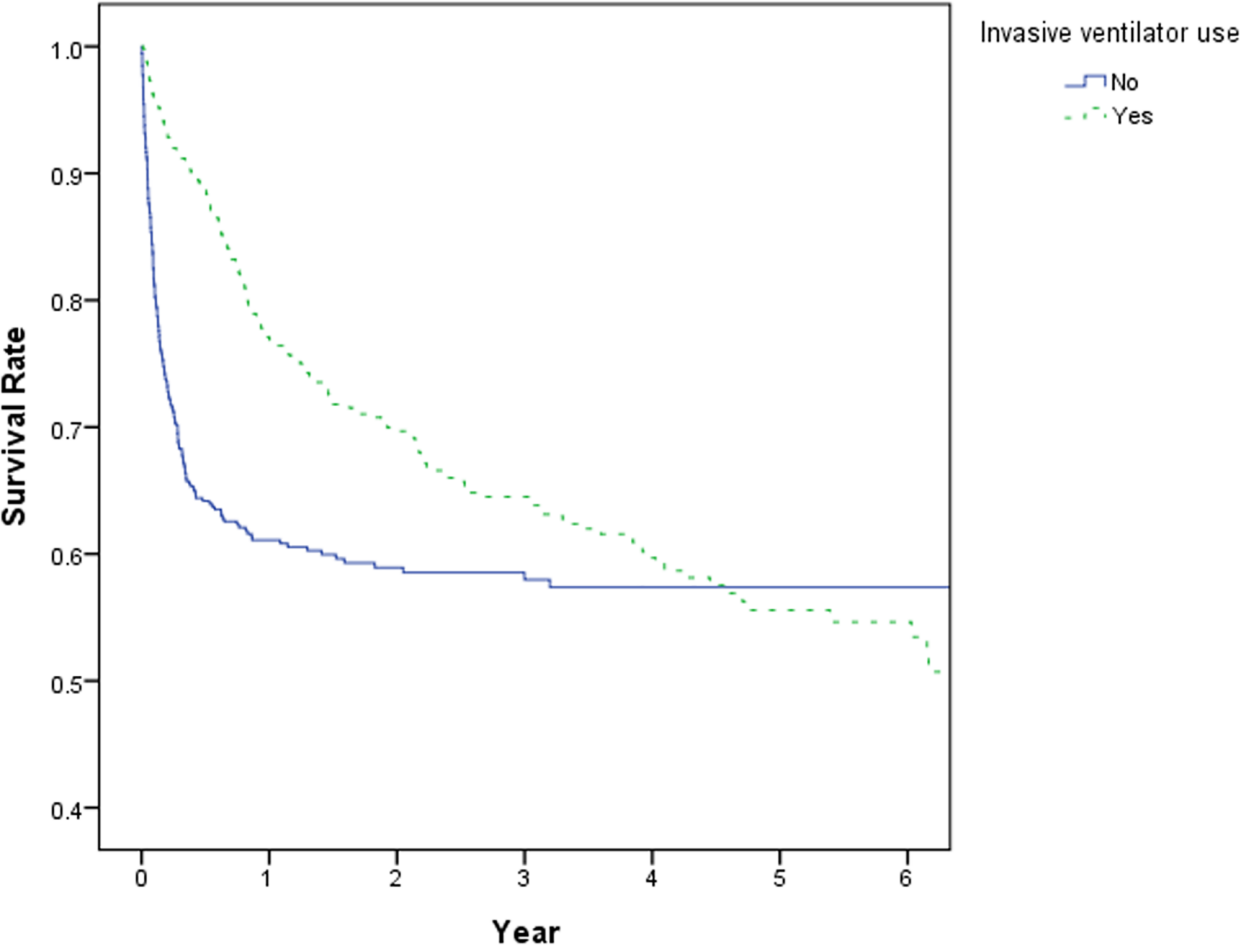
KM curves for the survival rates of the child and adolescent PMV patients according to their histories of invasive ventilation use.

A total of 1,019 PMV patients were sampled; however, 37.68% of them (384 patients) died by the end of 2011. A Kaplan—Meier curve (KM curve) was used to illustrate the survival rate of the child and adolescent PMV patients (Fig 1). The 1-year, 2-year, and 5-year mortality rates of these patients were respectively 32%, 37%, and 47%. Two KM curves were applied to depict the survival rate of the patients according to their histories of invasive ventilation use (Fig 2). The result revealed that the patients who had never used invasive ventilators attained a 1-year mortality rate of 40%, which was noticeably higher than that of those who had ever used invasive ventilators, and their 2-year mortality rate was approximately 42%. The 4-and-a-half-year mortality rate of all the PMV patients was 43% regardless of whether they had ever used invasive ventilators.

A Cox proportional hazards model was used to analyze the patient data. After the related variables were controlled, the relative risks and factors associated with the survival of the child and adolescent PMV patients were investigated. As listed in Table 1, the risk of mortality (ROM) of those aged 15–16 years was 0.65 times that of those aged <1 year (hazard ratio [HR] = 0.65; 95% CI: 0.44–0.96); the ROM of the patients with a CCI of ≥2 was1.99 times that of those with a CCI of 0 (HR = 1.99; 95% CI: 1.46–2.72); the ROM of the patients diagnosed primarily with musculoskeletal diseases was 0.54 times that of those diagnosed primarily with respiratory diseases (HR = 0.54; 95% CI: 0.35–0.83); and the ROM of the patients who had used an invasive ventilators for ≦4–5 years was 0.65 times that of those had not used invasive ventilators (HR = 0.65; 95% CI:0.52–0.81). However, when the patients had used an invasive ventilator for more than 4.5 years, the ROM was not significantly different from that of the patients without an invasive ventilator (P>.05)

**Table 1:** Factors that affected the mortality of the child and adolescent PMV patients (N = 1,019).

Variables	N	%	Adj. HR	95% CI		P-value
**Total**	1019	100				
**Sex**						
Female (Ref.)	426	41.81	1.0			
Male	593	58.19	0.84	0.69	1.04	0.102
**Age**						
<1 y (Ref.)	400	39.25	1.0			
1–4 y	225	22.08	1.09	0.82	1.44	0.553
5–9 y	123	12.07	0.98	0.70	1.38	0.918
10–14 y	155	15.21	0.90	0.65	1.24	0.519
15–16 y	116	11.38	0.65	0.44	0.96	0.032
**Monthly Income of Parents**						
Low-Income Household (Ref.)	44	4.32	1.0			
≦17,280	69	6.77	1.55	0.81	2.97	0.184
17,281~22,800	329	32.29	1.66	0.95	2.89	0.075
22,801~28,800	223	21.88	1.49	0.84	2.63	0.175
28,801~36,300	109	10.70	1.66	0.89	3.07	0.109
36,301~45,800	89	8.73	1.00	0.52	1.93	0.994
45,801~57,800	86	8.44	1.11	0.58	2.13	0.760
≧57,801	70	6.87	1.11	0.56	2.20	0.773
**Urbanization Level of residence area**						
Level 1 (Ref.)	320	31.40	1.0			
Level 2	307	30.13	1.14	0.87	1.48	0.343
Level 3	179	17.57	1.08	0.79	1.48	0.635
Level 4	130	12.76	1.38	0.99	1.91	0.055
Level 5–7	83	8.15	1.24	0.83	1.84	0.295
**CCI**						
0(Ref.)	687	67.42	1.0			
1	229	22.47	1.15	0.89	1.48	0.294
≧2	103	10.11	1.99	1.46	2.72	<0.001
**Diagnosed Disease Prompting Ventilation**						
Respiratory (Ref.)	627	61.53	1.0			
Central Nervous System	138	13.54	1.09	0.81	1.47	0.573
Musculoskeletal	99	9.72	0.54	0.35	0.83	0.005
Other	155	15.21	0.99	0.73	1.36	0.958
**Invasive Ventilation Use**						
No (Ref.)			1.0			
≦4.5 years			0.65	0.52	0.81	<0.001
>4.5 years			2.90	0.36	23.76	0.320

CI: confidence interval; Adj. HR: adjusted hazard ratio; CCI: Charlson Comorbidity Index.

Generalized estimating equations were employed for the multiple regression analysis on the average annual medical service uses of the patients (i.e., outpatient visits, emergency services, and hospitalization). Each of the patients spent an average of NT$948,645 annually (Table 2) on medical expenses. The results revealed that the ages of the patients, the diagnosed diseases prompting their ventilation, and their histories of invasive ventilation use were significantly correlated with their average annual medical expenses (P < .05). The average annual medical expense of the patients aged 1–4 years (NT$1,081,060/year) was higher than that of the patients aged 15–16 years (NT$654,726/year). The average annual medical expenses of the patients who received ventilation for respiratory diseases (NT$959,431/year), those that received ventilation for central nervous system diseases (NT$1,179,609/year), and those for musculoskeletal diseases (NT$1,019,304/year) were all higher than that of those that received ventilation for other diseases (NT$555,861/year). The average annual medical expense of the patients who had used invasive ventilators (NT$1,232,201/year) was higher than that of those who had not used invasive ventilators (NT$557,830/year).

**Table 2:** Average annual medical expense of the child and adolescent PMV patients and the associated factors (n = 547).

Variable	N	Mean	SD	P-value^a^	β	SE	P-value
**total**	547	948,645	799,004				
**Sex**							
Female	212	999,084	800,090	0.241			
Male	335	916,725	797,863		-64,236	65,721	0.329
**Age**				0.008			
<1 y	199	998,177	856,554				
1–4 y	131	1,081,060	903,215		-125,759	87,585	0.152
5–9 y	67	898,729	690,389		-225,089	107,860	0.037
10–14 y	85	892,714	676,223		-196,714	98,205	0.046
15–16 y	65	654,726	538,296		-376,635	116,948	0.001
**Monthly Income of Parents**				0.335			
Low-Income Households	30	1,119,495	563,286				
≦17,280	46	1,008,962	893,215		-112,537	172,893	0.515
17,281~22,800	154	893,427	776,037		-142,694	146,430	0.330
22,801~28,800	122	1,023,546	868,513		-95,172	150,420	0.527
28,801~36,300	52	964,562	848,436		-170,903	170,462	0.317
36,301~45,800	56	971,292	849,782		-227,067	166,989	0.175
45,801~57,800	50	941,477	782,792		-186,840	169,748	0.272
≧57,801	37	671023	486,057		-419,306	182,042	0.022
**Urbanization Level of residence area**				0.125			
Level 1	169	958,774	765,147				
Level 2	162	923,936	756,252		29,096	82,028	0.723
Level 3	108	1,096,122	974,718		105,231	90,768	0.247
Level 4	64	873,842	794,925		-60,856	108,357	0.575
Level 5–7	44	747,524	529,705		-143,521	128,394	0.264
**CCI**				0.266			
0	375	924,681	784,738				
1	128	1,045,779	874,088		104,552	77,647	0.179
≧2	44	870,305	673,962		24,701	123,210	0.841
**Diagnosed Disease Prompting Ventilation**				<0.001			
Respiratory	331	959,431	816,913				
Central Nervous System	82	1,179,609	670,830		78,700	92,376	0.395
Musculoskeletal	65	1,019,304	873,363		-6,152	99,850	0.951
Other	69	555,861	638,192.		-133,834	107,092	0.212
**Invasive Ventilation Use**				<0.001			
No	230	557,830	461,618				
Yes	317	1,232,201	869,954		602,939	71,856	<0.001

^a^ t test or ANOVA test.

SD: standard deviation; CCI: Charlson Comorbidity Index

Controlling for other variables revealed significant correlations between age, monthly income of parents, and status of invasive ventilation use (P < .05). First, participants who were aged 5–9 years (NT$225,089), 10–14 years(NT$196,714), and 15–16 years(NT$376,635) spent significantly less on medical expenses compared with those who were aged less than 1 year (P < .05). Second, the participants whose parents had a monthly income ≥NT$57,801 spent significantly less on average annual medical expenses (NT$419,306; P < .05) than did those from low-income households. Finally, the average annual medical expenses of participants who had used invasive ventilators was significantly higher than that of those who had not used invasive ventilators (NT$602,939; P < .05).

Table 3 lists the average annual medical service use of the child and adolescent PMV patients. Per patient, the average annual number of outpatient visits was 32.1 times per year; the average number of emergency services received was 1.6 time per year; and the average number of days of hospitalization was 160.8 days per year. Per patient, the average annual medical expense for hospitalization (NT$821,703/year) was the highest, followed sequentially by that of outpatient visits (NT$123,136/year), and that of emergency services (NT$3,806/year). These indicated that the average annual total medical expense of the child and adolescent PMV patients was NT$948,675 per year.

**Table 3:** Average annual medical service uses and expenses of the child and adolescent PMV patients (n = 547).

Medical Service Uses	Mean	SD	Min	Max	Median
**Outpatient Visits**	32.1	25.4	0.0	154.3	28.9
Expense	123,137	120,934	0.0	610,432	82,307
**Emergency Services**	1.6	2.2	0.0	20.6	0.7
Expense	3,806	5,263	0.0	32,930	1,880
**Days of Hospitalization**	160.8	190.4	0.0	888.5	72.7
Expense	821,703	834,012	0.0	4,971,166	497,394
**Total Annual Expense**	948,645	799,004	0.0	5,158,408	695,904

SD: standard deviation

## Discussion

The ROM of the patients aged 15–16 years was 0.65 times that of those aged <1 years (HR = 0.65). These results are different from the findings of previous studies on adult PMV patients, in which the ROM increased with age [7, 16]. The lower ROM the patients aged 15–16 years observed in the present study might be attributable to how they were physically more developed than those aged <1 years. The mortality rate of the patients with a CCI of 2 was twice as high as that of those with a CCI of 0 (HR = 2.0), revealing that the mortality rate rose with the CCI. This is consistent with the findings in previous studies, in which PMV patients had higher chances for survival, the lower that their CCI was regardless of their age [17, 18, 19]. The mortality rate of the patients who had received ventilation for musculoskeletal diseases was 0.54 times that of those who had received ventilation for respiratory diseases (HR = 0.54). These results are consistent with the findings in previous studies, in which the death of child PMV patients was primarily caused by chronic respiratory failure [20, 21]. The mortality rate of the patients who had used invasive ventilators was 0.66 times that of those who had not used invasive ventilators (HR = 0.66). This finding contradicts previous research results in Canada, in which the mortality rate of the child and adolescent PMV patients who had used invasive ventilators was 26%, and that of those that had not used invasive ventilators was 13% [21]. This inconsistency is caused by how the medical expenses are fully paid by the NHI Administration for PMV patients who are hospitalized; thus, the patients receiving invasive ventilation are more willing to stay in the hospitals than those not receiving invasive ventilation, and the quality of their medical care is therefore satisfactory. Consequently, the mortality rate of the patients who had used invasive ventilators was determined to be lower than that of those who had not used invasive ventilators. Previous studies in Taiwan focusing on adult PMV patients have reported that the mortality rate of the patients who used noninvasive ventilators was 48%, and that of those who used invasive ventilators was 28% [22], consistent with the findings in this study.

The economic (monthly income of parents) and environmental (urbanization level of residence area) factors did not significantly influence the mortality rate of the child and adolescent PMV patients. This was because the economic burdens of the PMV patients diagnosed with severe respiratory catastrophic injuries and illness were partially mitigated by the NHI program [13], thus eliminating the requirement of their parents to pay additional medical expenses. Additionally, because of the proximity of most residences to hospitals in Taiwan, the varying urbanization levels of the living environment did not cause Taiwanese people relevant inconvenience in seeking medical services.

Per patient, the average annual number of days of hospitalization was 160.8 days per year; and the average annual hospitalization expense was NT$821,000 per year. Previous studies have indicated that, per adult PMV patient, the average annual number of days of hospitalization was 102 days per year [23]; and the average annual hospitalization expense was NT$764,000 per year [24]. These results revealed that the average annual number of days and expenses of hospitalization of child and adolescent PMV patients was higher than those of adult PMV patients.

The average annual medical expense of the PMV patients aged ≧5 years was significantly lower than that of those aged <1 years. These results are consistent with those of previous studies that indicated that almost 50% of the medical expenses of child and adolescent PMV patients are spent on patients aged <1 years [17]. The average annual medical expenses of patients with parents who earn ≥NT$57,801 per month was lower than that of the patients from low-income households. This was because the PMV patients received partial mitigation for the medical expenses related to severe respiratory injuries or diseases, and the medical wards were exempt from ward charges, thus motivating both patients with high incomes and those with low incomes to stay in hospitals and prolong medical service use. The average annual medical expense of the patients who used invasive ventilators was higher than that of those who did not use invasive ventilators. These results are consistent with the findings in previous studies, in which the medical expenses of PMV patients who used invasive ventilators were higher than those of the patients who did not use invasive ventilators [25].

## Conclusion

This study determined that the factors affecting the survival of child and adolescent PMV patients are their age, their comorbidity, the diagnosed diseases prompting their ventilation, and the status of invasive ventilation use. The factors affecting the average annual medical expense of these patients are their age, economic factors, and status of invasive ventilation use. The statuses and factors of the medical service use and survival of the child and adolescent PMV patients in Taiwan can provide a reference for the NHI Administration when integrating and piloting PMV-related programs in the future.

## References

[1] National Health Insurance Administration, Ministry of Health and Welfare. The National Health Insurance Statistics, 2014. Part B. Statistical Tables II. Statistics 2014(Disc Data) 4. Medical Benefits. http://www.nhi.gov.tw/English/webdata/webdata.aspx?menu=11&menu_id=296&WD_ID=296&webdata_id=4835

[2] Estenssoro E., González F., Laffaire E., Canales H., Sáenz G., Reina R., et al (2005). Shock on admission day is the best predictor of prolonged mechanical ventilation in the ICU. CHEST Journal, 127(2), 598–603. 10.1378/chest.127.2.598 15706002

[3] Lone N. I., & Walsh T. S. (2011). Prolonged mechanical ventilation in critically ill patients: epidemiology, outcomes and modelling the potential cost consequences of establishing a regional weaning unit. Crit Care, 15(2), R102 doi: 10.1186/cc10117 21439086 10.1186/cc10117 PMC3219374

[4] MacIntyre N. R., Epstein S. K., Carson S., Scheinhorn D., Christopher K., & Muldoon S. (2005). Management of patients requiring prolonged mechanical ventilation: report of a NAMDRC consensus conference. CHEST Journal,128(6), 3937–3954. 10.1378/chest.128.6.3937 16354866

[5] Damuth E., Mitchell J. A., Bartock J. L., Roberts B. W., & Trzeciak S. (2015). Long-term survival of critically ill patients treated with prolonged mechanical ventilation: a systematic review and meta-analysis. The Lancet Respiratory Medicine, 3(7), 544–553. doi: 10.1016/S2213-2600(15)00150-2 26003390 10.1016/S2213-2600(15)00150-2

[6] National Health Insurance Administration, Ministry of Health and Welfare (2012) Business Implementation Report—February. http://goo.gl/Js50GQ (in Chinese)

[7] Lin M. S., Yan Y. H., Wang J. D., Lu H. M., Chen L., Hung M. C., et al (2013). Improved survival for an integrated system of reduced intensive respiratory care for patients requiring prolonged mechanical ventilation. Respiratory care, 58(3), 517–524. doi: 10.4187/respcare.01530 22906762 10.4187/respcare.01530

[8] National Health Insurance Administration, Ministry of Health and Welfare(2014) Part A. Abstract and Statistical Analysis. The National Health Insurance Statistics, 2013. http://www.nhi.gov.tw/English/webdata/webdata.aspx?menu=11&menu_id=296&WD_ID=296&webdata_id=4645

[9] Hsia S. H., Lin J. J., Huang I. A., & Wu C. T. (2012). Outcome of long-term mechanical ventilation support in children. Pediatr Neonatol, 53(5), 304–308. doi: 10.1016/j.pedneo.2012.07.005 23084723 10.1016/j.pedneo.2012.07.005

[10] Wallis C., Paton J. Y., Beaton S., & Jardine E. (2010). Children on long-term ventilatory support: 10 years of progress. Archives of disease in childhood, 96(11), 998–1002. doi: 10.1136/adc.2010.192864 21109507 10.1136/adc.2010.192864

[11] Racca F., Bonati M., Del Sorbo L., Berta G., Sequi M., Capello E., et al (2011). Invasive and non-invasive long-term mechanical ventilation in Italian children. Minerva anestesiologica, 77(9), 892–901. 21878871

[12] National Health Insurance Administration, Ministry of Health and Welfare(2016). Universal Health Coverage in Taiwan. http://www.nhi.gov.tw/English/webdata/webdata.aspx?menu=11&menu_id=290&webdata_id=2974&WD_ID=290

[13] National Health Insurance Administration, Ministry of Health and Welfare (2013). Regulations Governing the Exemption of the National Health Insurance Beneficiaries from the Co-Payment. http://www.nhi.gov.tw/English/webdata/webdata.aspx?menu=11&menu_id=295&WD_ID=295&webdata_id=2431

[14] Liu C. Y., Hung Y. T., Chuang Y. L., Chen Y. J., Weng W. S., Liu J. S., et al (2006). Incorporating development stratification of Taiwan townships into sampling design of large scale health interview survey. J Health Manag, 4(1), 1–22.

[15] Deyo R. A., Cherkin D. C., & Ciol M. A. (1992). Adapting a clinical comorbidity index for use with ICD-9-CM administrative databases. Journal of clinical epidemiology, 45(6), 613–619. 1607900 10.1016/0895-4356(92)90133-8

[16] Pilcher D., Bailey M., Treacher D., Hamid S., Williams A., & Davidson A. (2005). Outcomes, cost and long term survival of patients referred to a regional weaning centre. Thorax, 60(3), 187–192. doi: 10.1136/thx.2004.026500 15741433 10.1136/thx.2004.026500 PMC1747325

[17] Benneyworth B. D., Gebremariam A., Clark S. J., Shanley T. P., & Davis M. M. (2011). Inpatient health care utilization for children dependent on long-term mechanical ventilation. Pediatrics, 127(6), e1533–1541. doi: 10.1542/peds.2010-2026 21576303 10.1542/peds.2010-2026 PMC3103275

[18] Dermot F. J., Sansone G. R., Shakya K., & Kaner R. J. (2014). Prolonged Mechanical Ventilation in 540 Seriously Ill Older Adults: Effects of Increasing Age on Clinical Outcomes and Survival. Journal of the American Geriatrics Society, 62(1), 1–9. doi: 10.1111/jgs.12597 24404850 10.1111/jgs.12597

[19] Baldwin M. R., Narain W. R., Wunsch H., Schluger N. W., Cooke J. T., Maurer M. S., et al (2013). A prognostic model for 6-month mortality in elderly survivors of critical illness. Chest, 143(4), 910–919. doi: 10.1378/chest.12-1668 23632902 10.1378/chest.12-1668 PMC3616685

[20] Edwards J. D., Kun S. S., & Keens T. G. (2010). Outcomes and causes of death in children on home mechanical ventilation via tracheostomy: an institutional and literature review. The Journal of pediatrics, 157(6), 955–959.e952. doi: 10.1016/j.jpeds.2010.06.012 20713294 10.1016/j.jpeds.2010.06.012

[21] McDougall C. M., Adderley R. J., Wensley D. F., & Seear M. D. (2013). Long-term ventilation in children: longitudinal trends and outcomes. Arch Dis Child, 98(9), 660–665. doi: 10.1136/archdischild-2012-303062 23838128 10.1136/archdischild-2012-303062

[22] Tung H. P., & Wu H. P. (2009). Clinical Value of Non-invasive Positive Pressure Ventilators in Patients with Acute Respiratory Failure. Journal of Emergency Medicine, Taiwan, 11(2), S14–S19.

[23] National Health Insurance Administration, Ministry of Health and Welfare (2006). Established ventilator-dependent Integrated Delivery System.: http://www.areahp.org.tw/upload/project/txt/951108A.ppt

[24] National Health Insurance Administration, Ministry of Health and Welfare. The National Health Insurance Statistics, 2010. Part A. Abstract and Statistical Analysis III. Statistical Analysis 4. Medical Benefits. http://www.nhi.gov.tw/English/webdata/webdata.aspx?menu=11&menu_id=296&WD_ID=296&webdata_id=4010

[25] Fakharian A., & Hill N. S. (2013). NIPPV: Where Are We Now? Tanaffos, 12(3), 6 25191466 PMC4153252

